# Parental Style, Dating Violence and Gender

**DOI:** 10.3390/ijerph16152722

**Published:** 2019-07-30

**Authors:** María Muñiz-Rivas, María Vera, Amapola Povedano-Díaz

**Affiliations:** 1 Department of Social Anthropology, Seville University, 41013 Seville, Spain; 2 Department of Education and Social Psychology, Pablo de Olavide University, 41013 Seville, Spain

**Keywords:** parenting style, dating violence, online, offline, gender

## Abstract

The relationship between parenting styles and teen dating violence has become a relevant research topic in recent years, especially related to violence inflicted online. To more fully understand this relationship, the objective of the present study was to examine which parenting style (authoritarian, indulgent, authoritative, or neglectful) best protects against dating violence in adolescent relationships. A total of 1132 adolescents of both sexes participated in this study (46.4% boys and 53.6% girls), with ages between 14 and 18 years old (M = 15.6, SD = 1.3). A multivariate factorial design was applied (MANOVA, 4 × 2), using the parenting style, the parents’ gender, and the adolescents’ gender as independent variables, and the dating violence dimensions (online and offline) as dependent variables. As the results show, the lowest scores on all the dating violence dimensions examined were obtained by adolescents from indulgent families. In addition, three interaction effects were observed between the mother’s parenting style and the adolescent’s gender on online violence (e-violence and control), and the father’s parenting style on offline violence (verbal-emotional). Thus, adolescents with authoritarian mothers obtained the highest scores on violence and control inflicted online, respectively, and adolescent girls with authoritarian fathers obtained the highest scores on verbal-emotional violence. These findings suggest that the indulgent style is the parenting style that protects against violence in teen dating relationships, and they also highlight the risks of the authoritarian style as a family child-rearing model.

## 1. Introduction

After decades of silence, recent investigations have included the study of the prevalence of the use of violent behaviors to resolve conflicts in adolescent dating relationships [1,2]. For example, a meta-analysis of more than 100 studies revealed dating violence prevalence rates ranging from 5% to 90% in some studies [3,4]. The magnitude of these differences could indicate that the cases of dating violence vary in different contexts and countries. However, these discrepancies are probably due to different operationalizations of the construct and/or other questions related to the measurement instruments’ design. Despite these inconsistencies in the type of measurement used, there is a strong consensus in the scientific community that dating violence has serious consequences for the victims’ physical and psychological health [1,5,6]. Dating violence refers to repeated and abusive behaviors used by adolescent boyfriends toward their girlfriends or ex-girlfriends in order to have dominion and control over them and the relationship, usually in situations where they are not living together [7].

In recent years, with the development of Information and Communication Technologies (ICTs), dating violence has also been carried out through the Internet and virtual social networks [8,9]. We define online dating violence as behaviors expressed in the form of threats, insults, or ridicule through comments or images online that can harm the victim and control her environment (for example, control over the circle of friends or membership in an online group) [10]. Online dating violence develops through the repetition of behaviors of dominance, submission, and isolation over time [11]. It has often been associated with offline behaviors of physical and psychological violence, and so some authors have suggested that offline violence might be a precursor of online violence and vice versa [12]. In this regard, various studies indicate that there is often continuity between the relational spaces in this type of violence, such that the adolescent’s problems in the offline context move to the Internet and continue there [13,14]. Recent studies on online dating violence have included an analysis of its incidence [15,16,17]). As in the case of offline violence, discrepancies are found regarding its prevalence, with rates ranging from 15% [18] to 47.5% [17,19,20]. Moreover, these studies show a higher rate of this type of violence by boys than by girls.

In order to understand and prevent violence in teen dating relationships, many studies have focused on the analysis of factors related to its development in the first couple relationships [6,11]. The family is the most important socializing agent in a person’s life because it is the first environment where the identity develops and where the individual relates to others, and it is also the first nexus between the individual and the society in which s/he lives. Studies have shown that parents, through modeling and the reinforcement of certain behaviors (type of communication, violent behaviors, physical punishment, parental control, etc.), transmit a behavioral style to their children that adolescents often reproduce in their affective relationships [21,22,23], imitating violent behaviors and attitudes already present in the family [24]. In addition, these parental practices have been associated with serious behavioral and psychosocial adjustment problems in adolescents, variables that are closely linked to violence in couple relationships [25]. By contrast, the behaviors and attitudes that represent healthy family functioning (cohesion, support, and positive communication) are related to good psychological and emotional development in the children [26,27].

Based on the two-dimensional parental socialization model [28,29,30,31,32], two independent dimensions have been defined: acceptance/involvement and severity/imposition. Depending on the parents’ use of the educational practices that characterize these two dimensions, four parenting styles can be differentiated: authoritarian, high severity/imposition and low acceptance/involvement; indulgent, low use of strictness/imposition and high use of acceptance/involvement; authoritative, high acceptance/involvement and high strictness/imposition; and neglectful, low strictness/imposition and low acceptance/involvement [33,34].

The authoritative and indulgent parenting styles are characterized by the use of parenting practices related to warmth and rational communication. Traditionally, the authoritative style, which includes imposition and rigor practices as well as affective and rational practices, has been considered the ideal parenting style. However, recent studies in Spain and some countries in Europe and Latin America [35,36] point out that the indulgent parenting style is associated with similar or even better adolescent adjustment than the authoritative style [29,37,38,39]. Despite the relevance of these results, they should be interpreted with caution because they represent emerging research.

By contrast, authoritarian and neglectful parenting, both defined by less use of warmth and reasoning, tend to be associated with poor adaptation in adolescence. Some of the psychosocial adjustment indicators analyzed in these studies have been, for example, self-esteem, self-control, emotional distress, school achievement, the use of learning strategies, the use of substances, or behavior problems [27,30,32].

The family socialization process also includes aspects related to gender. Specifically, gender socialization refers to the process through which people begin to feel, think, and act differently depending on whether they are male or female, and it develops from early childhood [7]. During adolescence, the family plays an even more important role in gender socialization than in other stages of development, through the assimilation and reproduction of behaviors, attitudes, and roles associated with gender. Few studies have included the gender perspective in the study of parenting styles, and their results seem contradictory. Some studies have pointed out that the relationship between the four parenting styles and the children’s psychosocial development does not vary depending on the adolescent’s gender [33,40,41]. whereas other studies have shown gender differences in this process [42,43]. For example, some studies have pointed out that boys often receive more disciplinary actions and punishments than girls, which are related to a more authoritarian educational style [42,44], and these practices are probably related to a greater use of violence, as some studies suggest [45,46]. However, girls receive much more supervision and control than boys, which influences the development of their autonomy and confidence in decision-making [47].

Regarding the gender perspective in dating relationships, again the results are controversial. On the one hand, there are clear indications that violence in teen dating relationships is reciprocal [48,49]. On the other hand, many studies have shown that boys are more violent than girls [5,50], and that boys use and justify violence more [51,52]. Moreover, girls make greater use of reactive violence, whereas boys use more proactive violence as a form of control [50,53]. One possible interpretation is related to the degree to which both boys and girls incorporate society’s chauvinistic attitudes and beliefs and reproduce traditional gender roles where boys are strong and violent and girls are caregivers and submissive [54,55].

In fact, not only is gender learned through socialization, but violence is also learned this way. Thus, studies have found that boys’ greater involvement in forms of physical aggression could be attributed to learning “gender differentiated patterns of violence” [56]. Furthermore, some family-related risk factors are also linked to dating abuse, including: parental punitive practices, the lack of affective cohesion, frequent conflicts, inadequate family communication patterns, violent marital relations, and physical or sexual abuse of children by their parents. Previous studies [57] found that adolescents from families with indulgent socialization styles report the lowest levels of dating violence, whereas adolescents from families with authoritarian styles are more likely to become involved in violent relationships, followed by adolescents from families with a neglectful style. In other words, parenting styles based on affect, comprehension, and support are the most favorable for building a healthy adolescent relationship, particularly the mother’s style.

Despite the research interest in young people’s use of the Internet and virtual social networks, studies on parenting styles and online dating violence are still scarce. In spite of this gap, studies carried out on cyberbullying can provide some ideas about the relationships between the variables of interest in this research. For example, a study on cyberbullying with a Spanish sample shows that the indulgent parenting style, characterized by practices of acceptance and participation, is the most protective style in all the results analyzed, and is associated with the lowest levels of cyberbullying. By contrast, authoritarian parenting, characterized by rigor and imposition, is associated with the highest levels of cyberbullying [35].

Consequently, considering the previous research, the analysis of the family socialization model would provide us with broader knowledge about the way parenting styles influence online and offline dating violence in adolescence. Thus, this study proposes to establish a link between previous studies on the family’s influence in adolescence and the violence inflicted in dating relationships in this stage. Moreover, the objective of the present study is to analyze the relationship between the parenting styles and offline and online dating violence, focusing on the gender of the parents and the adolescents.

## 2. Method

### 2.1. Sample and Procedure

The sample was composed of 1132 teenagers enrolled in four schools in Andalucía (Spain). They were adolescents between 14 and 18 years old (*M* = 15.6, *SD* = 1.3); 46.4% were boys, and 53.6% were girls. All of them indicated currently having or having had a romantic partner in the past year. The average of the missing data obtained was 0.8%, and never more than 0.9% for an individual item. Therefore, estimations of the expected values in the general population are accurate [58].

Regarding the procedure, once the schools had been randomly selected, the director was contacted. With the director’s help, we held an informative seminar where the project was explained to teachers and parents. In this seminar, we answered any questions, and we asked for their collaboration. In all cases, permission was given by the parents. The questionnaire was administered to the adolescents in their usual classrooms during a regular class period. Adolescents were informed that participation was voluntary and confidential. The study met the ethical values described in the Declaration of Helsinki [59].

### 2.2. Instruments

Socialization style was measured with ESPA29 Parental Socialization Scale in Adolescence [28,31,39,60]. This instrument assesses, through 232 items, the parenting socialization styles of the two parents in different natural scenarios representative of everyday family life in Western culture. A child separately rates the behavior of his/her father and his/her mother in 29 significant situations; 16 refer to children’s behavior that conforms to family norms (e.g., “He/She respects the rules established in my house”), and 13 refer to behavior that goes against these rules (e.g., “He/She is dirty and messy”). For all 29 situations, the questionnaire obtains a global mean for each parent in the dimensions of acceptance/implication and strictness/imposition (similar to those of demandingness and responsiveness). From the scores on the two dimensions, the socialization style of each parent is classified as authoritative, indulgent, authoritarian or neglectful. All the items have a Likert response scale ranging from 1 (“Never”) to 4 (“Always”). Finally, internal consistency of acceptance/implication was 0.95 for the mother and 0.95 for the father; for strictness/imposition, it was 0.93 for the mother and 0.93 for the father.

E-dating violence was measured by the Couple´s Violence in Social Networks Scale in Adolescents (e-VPA) [10]. We evaluated the violent behavior exhibited towards the partner and former partners through the Internet. The scale consists of 20 items rated on a Likert-type scale with four response options ranging from 1 (“Never”) to 4 (“Always”). It is composed of two dimensions: (1) E-violence, consisting of four items measuring threats, insults, and public humiliation through online comments or images (example: “I have publicly threatened my boyfriend/girlfriend on his/her social network or done so by private message”) (α = 0.80). (2) E-control, consisting of six items evaluating the possessiveness related to the circle of friends or membership in a virtual group (example: “I get angry if my boyfriend/girlfriend is in a photo with people I don’t like”) (α = 0.86).

Dating violence was measured by the Conflict in Adolescent Dating Relationships Inventory (CADRI) [61], validated in Spanish by Fernández-Fuertes, Fuertes, and Pulido [62]. This inventory consists of 32 items rated on a Likert-type scale with four response options ranging from 1 (“Never”) to 4 (“Always”), and evaluates violent behavior toward the partner. It is composed of three dimensions: (1) Relational violence, with two items that evaluate control and social isolation induction (example: “I spread false rumors about him/her”) (α = 0.64); (2) verbal-emotional violence, with 10 items that evaluate humiliation, intimidation, insults and threats, or any other means that affect emotional stability (example: “I said something just to make him/her angry”) (α = 0.83); and (3) physical violence, containing four items that evaluate any action that affects the adolescent´s integrity by causing physical damage or suffering (example: “I pushed/shook him/her”) (α = 0.79).

### 2.3. Data Analysis

First, the prevalence of violence in the sample is presented. Second, percentages, descriptive analyses, correlations, and Cronbach´s alpha were carried out among all the variables under study. Third, two multivariate analyses of variance (MANOVAs) (4 × 2) were performed to identify differences in violence, online and violence offline, depending on the mother’s and father´s parenting styles and gender and their interaction. All the analyses were carried out using SPSS 25. Univariate F follow-up tests were conducted for the multivariate significant overall differences, and significant results on the univariate tests were followed by Bonferroni’s comparisons of all possible pairs of means. We applied the same traditional design and robust statistical analyses as in other seminal studies (i.e., [30]).

## 3. Results

Table 1 shows the prevalence of violence in the sample. In order to find the highest scores, we calculated the mean + 1SD for each variable, and we included only those with high scores. Thus, the table presents the percentage and number of boys and girls whose scores on all the types of violence measured are among the highest.

Table 2 shows the numbers of cases in the parenting style groups, mean scores, and standard deviations on measures of parental dimensions (see Table 1)

The percentages of mothers and fathers in the four parenting style groups (neglectful, authoritarian, indulgent and authoritative) are quite similar: 28.8% versus 30.2%; 21.1% versus 19.6%; 21.9% versus 19.3%; and 28.2% versus 30.8%, respectively. The highest scores on acceptance/involvement for both are found in the authoritative style. Therefore, the lowest scores are found in the neglectful style. The same pattern occurs in the case of strictness/imposition; that is, the highest scores for both are found in the authoritative style because the lowest scores can be observed in the neglectful style.

Table 3 shows descriptive analyses, means, standard deviations, Cronbach´s alphas, and correlations between all the variables under study. Means, standard deviations, and all alphas are in the expected direction. Moreover, the correlations show that the acceptance/involvement and strictness/imposition dimensions have a powerful relationship between the mother and the father. Moreover, the mother´s strictness/imposition seems to be especially linked to the violence variables. Finally, all the dimensions of violence online and offline are positively and significantly related.

After conducting descriptive analyses, we performed two MANOVAs to find out if there were differences in violence, online as well as offline, depending on the parental socialization style and gender, and to test a possible interaction effect between these two variables. Table 4 shows the significant main and interaction effects of the two variables for mothers and the significant main and interaction effects of the gender variable. Table 5 shows the same effects for fathers. Nonetheless, because the interaction effect of the parenting style and gender is significant, it makes sense to continue.

Finally, univariate analysis of mothers’ (Table 6) and fathers’ (Table 7) styles as well as gender (Table 8) indicated how these significant differences are distributed among adolescents.

As Table 6 and Table 7 show, and regarding the main effect of parenting style, verbal-emotional violence has significant main effects in the case of both parents. In all cases, the highest scores on violence correspond to authoritarian parents and the lowest levels to indulgent parents. The mother´s style also shows significant main effects for e-violence and physical violence, and again the highest scores on violence correspond to authoritarian mothers and the lowest levels to indulgent mothers.

Regarding the main effect of the gender of the adolescents (see Table 8), significant effects appear on almost all the variables, specifically, on e-violence (higher in boys), e-emitted control (higher in girls), relational violence (higher in boys), and verbal-emotional violence (higher in girls).

**Figure 1:**
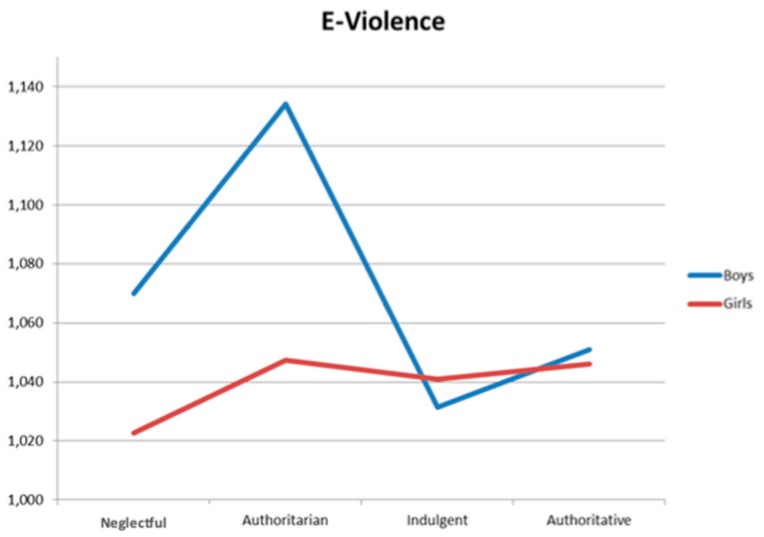
Iteration effect on e-violence of the mother´s style.

**Figure 2:**
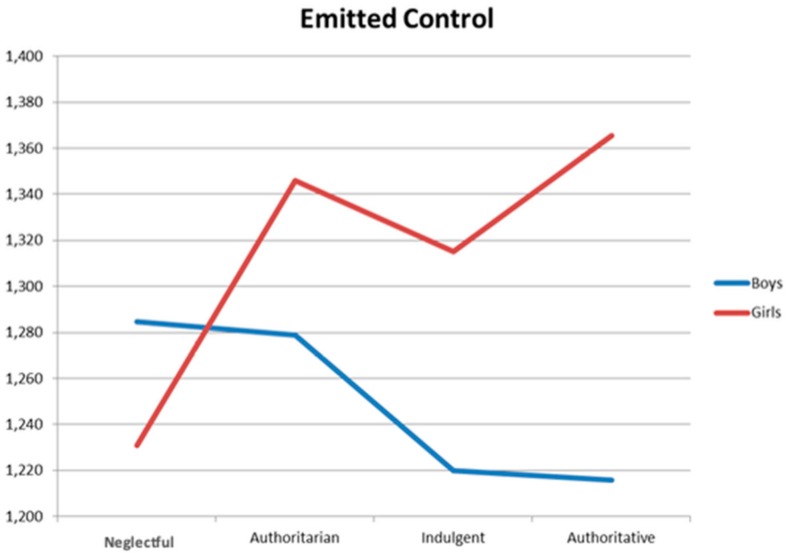
Iteration effect on e-emitted control of the mother´s style.

Finally, the interaction effect between parental socialization styles and adolescents´ gender on violence (online and offline) was examined. In the case of the mother´s style, significant differences appear in e-violence F(2, 1131) = 2.54, *p* < 0.050, and e-emitted control F(2, 1131) = 3.03, *p* = 0.029. Figure 1 and Figure 2 shows these results, respectively. The two interactions show a different pattern: Whereas in e-violence boys show higher values (peak in the authoritarian style), girls show similar values for the authoritarian, authoritative, and indulgent styles. In Figure 2, girls and boys show a completely opposite pattern in the neglectful style: whereas girls have the lowest levels of e-emitted control, boys have the highest. The opposite occurs in the authoritative style: girls have the highest levels of e-emitted control, and boys have the lowest.

**Figure 3:**
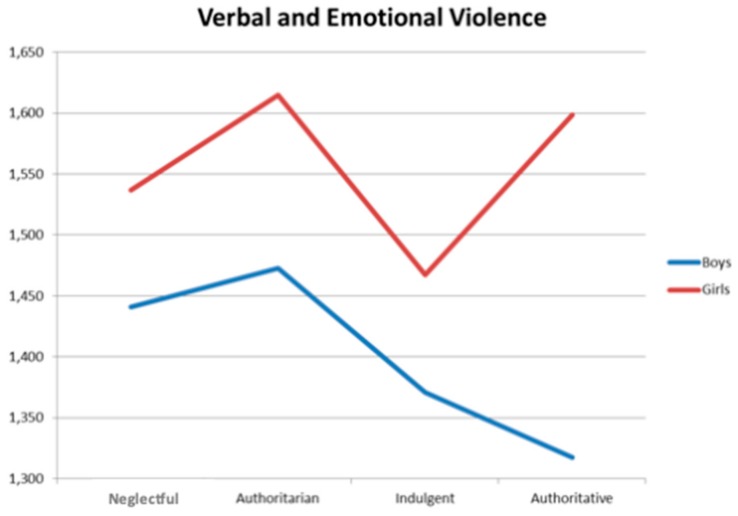
Iteration effect on verbal and emotional violence of the father´s style.

In the case of the father´s style, significant differences appear in verbal-emotional violence F(2, 1131) = 3.24, *p* = 0.024. Figure 3 shows these results. In the interaction, girls show higher values than boys, and both have their peak values in the authoritarian style. However, when the father is authoritative, it has a very different effect on boys and girls: whereas in girls the level of violence increases, in boys it decreases.

## 4. Discussion

The purpose of the present study was to analyze the relationship between parenting styles and adolescent dating violence from a gender perspective. In our study, the percentages in the four parenting style groups are similar to other studies in Spain [28,63,64]. In addition, our results are coherent with previous studies about the prevalence of e-dating and dating violence in Spain. They indicate that adolescent dating violence, in virtual and off-line contexts, is not an infrequent occurrence. Nearly 18% of the participants reported having been involved in some type of offline violence, and 12% of the sample reported having used violence and/or control online. In other words, boys and girls who establish their first couple relationships often resolve conflicts by using insults, threats, or coercion. These behaviors show poor management of the conflicts that arise naturally in human relationships and, especially, in dating relationships [65,66].

These results suggest that sons and daughters perceive few differences between the parenting styles of their mother and father. The highest scores on the difference in styles between the two parents were found in the authoritative style, and the lowest in the neglectful style, with the mother reaching higher levels of acceptance/involvement in both styles. These results agree with those obtained in previous studies pointing out that mothers, compared to fathers, are perceived as having greater influence, more unconditional acceptance, and more involvement with their sons and daughters [67,68]. One possible explanation for this result is that the mothers have greater exposure and involvement in daily child-rearing, specifically in areas related to affective relationships [69,70]. It is interesting to note that these differences between the two parents occur in both flexible parenting styles and more disciplinary parenting styles [69,71,72]. Although the authoritative style (strictness and warmth) seems to have benefits in some cultures to prevent and protect many internalizing and externalizing behaviors (i.e., the Anglo-Saxon context) [73,74], our results are consistent with a growing line of research that questions whether the authoritative parenting style is always associated with positive results in all cultures [28]. However, in European and Mediterranean countries, the indulgent parenting style (warmth but not strictness) provides many benefits to boys and girls. That is, children raised in families with indulgent styles showed equal or greater adjustment than authoritative households on variables such as the prevention of aggression and cyberaggression and traditional or cybernetic harassment [23,75], which is consistent with the results obtained in our study of online and offline dating violence.

In the case of offline and online dating violence, our data support the results obtained in other studies about the relationship between the severity/imposition of the mother and the possibility that the children will use violence in their couple relationships [76]. Various factors can explain these results, among them the influence of society’s messages about the feminine role and non-acceptance of the control and dominance women can exercise. These messages can lead male children to consider their mothers to be weak people with little power, and they can lead female children to try to distance themselves from this image of the vulnerable woman by using violence [77,78].

With regard to the relationship between the parenting style and dating violence, our results show statistically significant relationships in both parents. In all cases, the highest scores on both types of violence are related to authoritarian parents and the lowest to indulgent parents. However, there are some differences between mothers and fathers. For example, the mother’s parenting style has a strong relationship with the physical and verbal-emotional violence exercised by boys and girls in their offline and online dating relationships. These results are coherent with those found in previous studies showing a significantly higher probability of partner violence in adolescents educated with severe and imposing parenting styles, compared to young people whose parents use a more positive style [76,79]. In addition, some studies highlight the importance of the maternal parenting style in the correct psychological and social adjustment of their children [79,80]. Therefore, coercive practices such as firm control and strong discipline, as in the authoritarian style, may be more adverse than neglectful parenting. These results are consistent with other studies [81,82,83] that found that parental strictness, a characteristic of authoritarian and authoritative styles, is not an essential element for healthy psychological and social adjustment or to prevent violent attitudes in adolescence in different contexts.

Our results nuance the data found in some studies indicating that, in the virtual context, adolescents might be more influenced by their peers than by their parents [84,85]. However, our results highlight the parents’ importance and influence in online contexts, which is consistent with previous studies pointing out that, although the adolescent’s social network broadens and acquires greater importance as s/he develops, the family socialization environment continues to be extremely relevant and influential [86].

Moreover, our results showed interactions between the parenting style and the gender of the adolescents in the dating violence (online and offline) carried out. Thus, adolescent boys with authoritarian mothers have higher levels of online violence than girls, and lower levels than girls with indulgent mothers. However, in the control carried out online, the opposite occurs; adolescent girls raised in an authoritarian style tend to exercise more control over their partners than boys, whose parameters do not very much. These results coincide with previous studies that highlight the relationship between online violence and the lack of resources and strategies in adolescents from authoritarian families, especially the girls [60,72]. Moreover, boys and girls could react to the parent’s severity and imposition by becoming more involved in virtual spaces as a form of refuge, with the risk that incorrect use of these spaces can lead to using violence in online environments. This proposal must be confirmed in future studies.

The findings of the present study also indicate that girls whose parents are authoritarian tend to use more verbal-emotional violence than boys in the same circumstances. This may be due to the different gender socialization processes in childhood and adolescence, where female adolescents could be especially affected by the authoritarian parenting style and the related negative aspects in the family context [23,86]. Authoritarian parents usually punish girls who develop coping and conflict resolution resources and strategies by using coactive and imposing methods that can foment a special vulnerability to this socialization style in the family setting.

In summary, one of the most important contributions of the present study is the finding that indulgent parenting is the most consistent style for the prevention of violence in adolescent couples. This result is consistent with the third stage [87]. In the current digital era, the parental warmth dimension is enough to (1) support children when they display correct behavior; and (2) prevent children’s risk behaviors through reasoning and communicative practices. This result has also been obtained in most of the European countries that reinforce the idea of the person fitting in the context within a broader global framework. Undoubtedly, this study has some important limitations. In addition to the link to the parenting style, a more complete analysis of dating violence, both online and offline, would also require the consideration of the motivations that adolescent boys and girls attribute to violence, their perceptions of it, and their beliefs about romantic relationships. Moreover, this is a cross-sectional study that does not allow us to establish causal relationships, and so it would be necessary to perform longitudinal studies to better understand the way the parenting style affects violence in adolescent dating relationships. Finally, it is important to note that the effect sizes in the MANOVAs and ANOVAs are small. However, we are not concerned about validity because our results are congruent with recent studies that belong to the third stage [87] and with classical studies [38]. Thus, these results seem to maintain their importance in the field.

## 5. Conclusions

In conclusion, despite the limitations, the results of this study provide suggestive information about the relationship between the parenting style and teen dating violence, and they show the relationship between the two contexts (online/offline) in adolescents who perform this type of violence. First, the results indicate that there is a significant relationship between authoritarian and neglectful parenting styles and dating violence in adolescence [25,30]. Second, it is important to point out, perhaps as the main contribution of the present study, the results obtained for the interactions among the parenting style, dating violence (online and offline), and the adolescents’ gender. Thus, adolescents with authoritarian mothers obtained the highest scores on online violence (the boys on violence and the girls on control). Additionally, the father’s authoritarian style was also related to the highest scores on verbal-emotional violence (especially in the girls). By contrast, the lowest scores observed in all the dimensions of dating violence corresponded to the adolescents from families that used indulgent parenting. We think the findings of this study are especially important, given that the indulgent parenting style could be the most appropriate socialization style to prevent situations of violence in adolescent dating relationships (online and offline). Therefore, interventions designed to prevent and reduce dating violence should take the adolescents’ family context into account by analyzing, along with fathers and mothers, which parenting styles favor the healthiest couple relationships and the adequate psychosocial adjustment of boys and girls in offline and online contexts. Finally, the findings of the present study indicate the importance of taking the gender of both the parents and the adolescents into account. Although the present results should be viewed with caution, future studies are needed to continue the research in this suggestive line about the third stage. These new studies should also include gender as an important empirical variable that helps us to understand the relationships between parenting styles and dating violence in adolescence in offline and online contexts.

## Figures and Tables

**Table 1:** Violence prevalence among the sample (*N* = 1132).

	Total		Boys		Girls	
	%	*N*	%	*N*	%	*N*
e-violence	2.5%	28	3.6%	41	1.5%	17
e-emitted control	8%	91	10.1%	114	9.4%	106
Relational violence	4.7%	53	17.5%	198	3.1%	35
Verbal-emotional violence	6.5%	74	1.7%	19	10.5%	119
Physical violence	7.1%	80	37.7%	427	3.1%	35

**Table 2:** Means in the four categories and gender (*N* = 1132).

	Total		Neglectful		Authoritarian		Indulgent		Authoritative	
	M	F	M	F	M	F	M	F	M	F
Frequency	1132	1132	326	342	239	222	248	219	319	349
Percent	100	100	28.8	30.2	21.1	19.6	21.9	19.3	28.2	30.9
Acceptance/Involvement										
Mean	3.16	3.06	2.80	2.63	2.81	2.68	3.48	3.43	3.55	3.49
SD	0.45	0.52	0.33	0.39	0.31	0.36	0.22	0.23	0.21	0.28
Strictness/Imposition										
Mean	1.83	1.82	1.51	1.45	2.13	2.16	1.55	1.49	2.16	2.16
SD	0.40	0.44	0.21	0.22	0.31	0.32	0.21	0.21	0.28	0.32

Note. M = mother; F = father.

**Table 3:** Means, Standard Deviations, and Correlations (N = 1132).

	M	SD	α	1	2	3	4	5	6	7	8
1 Acceptance/Involvement M	3.16	0.45	0.82								
2 Strictness/Imposition M	1.83	0.40	0.93	0.16 (0.000)							
3 Acceptance/Involvement F	3.06	0.52	0.85	0.74 (0.000)	0.17 (0.000)						
4 Strictness/Imposition F	1.82	0.44	0.94	0.21 (0.000)	0.75 (0.000)	0.24 (0.000)					
5 e-violence	1.05	0.22	0.78	−0.07 (0.026)	0.07 (0.028)	−0.05 (0.115)	0.04 (0.195)				
6 e-emitted control	1.28	0.44	0.81	0.03 (0.302)	0.09 (0.004)	−0.01 (0.821)	0.03 0.271)	0.37 (0.000)			
7 Relational violence	1.09	0.31	*	−0.05 (0.127)	0.08 (0.005)	−0.05 (0.132)	0.07 (0.030)	0.37 (0.000)	0.22 (0.000)		
8 Verbal-emotional violence	1.51	0.64	0.81	−0.02 (0.595)	0.04 (0.196)	−0.06 (0.037)	0.04 (0.143)	0.20 (0.000)	0.30 (0.000)	0.43 (0.000)	
9 Physical violence	1.08	0.26	0.68	−0.03 (0.347)	0.07 (0.0229)	−0.03 (0.273)	0.05 (0.109)	0.31 (0.000)	0.26 (0.000)	0.44 (0.000)	0.43 (0.000)

Note: M = mother; F = father. *p* values are in brackets. * Cronbach´s alpha cannot be calculated because it only contains two items.

**Table 4:** MANOVA factorial (4^a^ × 2^b^) for adolescent dating violence (online and offline) for mothers (*N* = 1132).

Source of Variation	Λ	F	gl_between_	gl_error_	*p*
(A) Parenting Style ^a^	0.98	1.92	15	3078.43	0.017
(B) Gender ^b^	0.93	16.93	5	1115.00	<0.001
A × B	0.97	2.65	15	3078.43	0.001

Note: a^1^: neglectful, a^2^: authoritarian, a^3^: indulgent, a^4^: authoritative; b^1^: boy, b^2^: girl; *p* values are in brackets. Λ: wilks lambda; F: contrast statistic of MANOVA.

**Table 5:** MANOVA factorial (4^a^ × 2^b^) for adolescent dating violence (online and offline) for fathers (*N* = 1132).

Source of Variation	Λ	F	gl_between_	gl_error_	*p*
(A) Parenting Style ^a^	0.99	1.15	15	3078.43	0.302
(B) Gender ^b^	0.93	16.00	5	1115.00	<0.001
A x B	0.98	1.65	15	3078.43	0.054

Note: a^1^: neglectful, a^2^: authoritarian, a^3^: indulgent, a^4^: authoritative; b^1^: boy, b^2^: girl; *p* values are in brackets.

**Table 6:** Means, standard deviations (in brackets), F values, *p* values, effect size, and post hoc Bonferroni procedure for both parenting style groups across online and offline violence (*N* = 1132) in Mothers.

	Neglectful	Authoritarian	Indulgent	Authoritative	F(2, 1131)	*p*	*η* ^2^
e-violence	1.05 (0.01)	1.09 (0.01) *	1.04 (0.01) *	1.05 (0.01)	2.99	0.030	0.007
e-emitted control	1.26 (0.02)	1.31 (0.03)	1.26 (0.03)	1.29 (0.03)	0.84	0.472	0.003
Relational violence	1.08 (0.02)	1.11 (0.02)	1.06 (0.02)	1.11 (0.02)	1.91	0.127	0.005
Verbal-emotional violence	1.47 (0.02)	1.55 (0.03) *	1.41 (0.03) *	1.48 (0.03)	4.43	0.004	0.013
Physical violence	1.06 (0.01)	1.12 (0.02) *	1.05 (0.02) *	1.07 (0.01)	3.31	0.020	0.010

Note. * significant differences between groups according to the post hoc Bonferroni test.

**Table 7:** Means, standard deviations (in brackets), F values, *p* values, effect size, and post hoc Bonferroni procedure for both parenting style groups across online and offline violence (*N* = 1132) in Fathers.

	Neglectful	Authoritarian	Indulgent	Authoritative	F(2, 1131)	*p*	*η* ^2^
e-violence	1.07 (0.01)	1.07 (0.02)	1.03 (0.01)	1.05 (0.01)	2.07	0.102	0.005
e-emitted control	1.29 (0.02)	1.29 (0.03)	1.27 (0.03)	1.27 (0.02)	0.18	0.910	0.000
Relational violence	1.10 (0.02)	1.10 (0.02)	1.06 (0.02)	1.09 (0.02)	0.89	0.446	0.003
Verbal-emotional violence	1.49 (0.02)	1.54 (0.03) *	1.42 (0.03) *	1.46 (0.02)	3.29	0.020	0.009
Physical violence	1.08 (0.01)	1.09 (0.02)	1.04 (0.02)	1.07 (0.01)	1.83	0.141	0.005

Note. * significant differences between groups according to the post hoc Bonferroni test.

**Table 8:** Means, standard deviations (in brackets), F values, *p* values for gender across online and offline violence (*N* = 1132).

	Boys	Girls	F(1, 1131)	*p*	*η* ^2^
e-violence	1.07 (0.01)	1.04 (0.01)	5.01	0.025	0.005
e-emitted control	1.25 (0.02)	1.31 (0.02)	5.71	0.017	0.005
Relational violence	1.12 (0.01)	1.06 (0.01)	8.10	0.005	0.007
Verbal-emotional violence	1.40 (0.02)	1.55 (0.02)	33.24	<0.001	0.033
Physical violence	1.07 (0.01)	1.08 (0.01)	0.19	0.660	0.001
